# Serum Kisspeptin and Its Relation to Metabolic Parameters and Glucose Metabolism in Prepubertal and Pubertal Obese Children

**DOI:** 10.1155/2020/8826401

**Published:** 2020-11-10

**Authors:** Kochakorn Sithinamsuwan, Pat Mahachoklertwattana, Somboon Wankanit, Suwannee Chanprasertyothin, Sarunyu Pongratanakul, Patcharin Khlairit, Preamrudee Poomthavorn

**Affiliations:** ^1^Department of Pediatrics, Faculty of Medicine Ramathibodi Hospital, Mahidol University, Bangkok 10400, Thailand; ^2^Research Center, Faculty of Medicine Ramathibodi Hospital, Mahidol University, Bangkok 10400, Thailand

## Abstract

**Objective:**

Kisspeptin, a puberty control neuropeptide, has been discovered to have an additional role in metabolism and glucose homeostasis regulation. This study aimed to determine the association of serum kisspeptin with metabolic parameters and glucose metabolism in obese children. *Design, Patients and Measurements.* A cross-sectional study of 270 obese children was conducted. All children underwent an oral glucose tolerance test and had serum kisspeptin, glycated hemoglobin (HbA1c), and lipid profile measurements. Body fat mass was assessed by bioelectrical impedance analysis. Serum kisspeptin levels of both prepubertal and pubertal children with two HbA1c ranges, <5.7% (normal range) and 5.7–6.4% (prediabetes range), were analyzed and correlated with metabolic parameters and glucose metabolism status.

**Results:**

The median (IQR) serum kisspeptin level of only pubertal (not prepubertal) children with prediabetes HbA1c was higher than those with normal HbA1c (53.2 (33.9, 69.8) and 37.8 (29.6, 67.5) pg/mL; *p* = 0.015, respectively). There were no differences in serum kisspeptin levels among children with different glucose metabolism status. During pubertal progression, serum kisspeptin reached the highest level at Tanner stage II only in obese boys. Additionally, there was a positive correlation between serum kisspeptin and HbA1c after adjusting for puberty (*β* = 12.87; *p* = 0.001). No correlations between serum kisspeptin and insulin sensitivity indices, insulin secretion indices, lipid profile, blood glucose, as well as percentage of body fat were demonstrated.

**Conclusions:**

Serum kisspeptin levels in pubertal obese children with prediabetes HbA1c were greater than those with normal HbA1c. Serum kisspeptin was positively associated with HbA1c, but not with glucose metabolism status.

## 1. Introduction

Childhood obesity has been increasing worldwide. It is well-known to cause metabolic abnormalities, including abnormal glucose tolerance, diabetes mellitus, and dyslipidemia [1]. Pathogenesis of obesity involves a combination of excessive caloric intake, sedentary lifestyle, and the effects of multiple hormones and adipokines that involve body weight and appetite control such as leptin, adiponectin, proopiomelanocortin, prohormone convertase 1, melanocortin receptor 3 (MC3R), and melanocortin receptor 4 (MC4R) [2]. In the hypothalamus, kisspeptin is a neuropeptide produced by kisspeptin neurons. It plays a pivotal role in the control of pubertal onset by stimulating the secretion of gonadotropin-releasing hormone [3]. Recently, kisspeptin has been demonstrated to have a role in regulating energy balance and glucose metabolism [4–8]. The kisspeptin receptor (*Kiss1r*) gene knock-out female mice showed greater body weight, adiposity, fasting blood glucose, and the proportion of impaired glucose tolerance, but lower energy expenditure than the controls [5, 6]. Kisspeptin (*KISS1*) and *KISS1R* mRNAs were shown to be expressed in human and mouse islet cells [7]. Additionally, exogenous kisspeptin administration increased glucose-induced insulin secretion (GSIS) in both human and mouse islets [7], suggesting an important role of KISS1/KISS1R system in controlling islet function.

Adult studies of serum kisspeptin in relation to metabolic parameters and glucose metabolism have shown conflicting results. Several studies demonstrated a positive effect of kisspeptin on glucose metabolism. Negative association between the serum kisspeptin level and body mass index (BMI) was observed in women with polycystic ovary syndrome (PCOS), obese women, postmenopausal women, nondiabetic obese adults, and female adolescents with anorexia nervosa [9–14]. Additionally, the serum kisspeptin level was shown to be negatively associated with insulin resistance in PCOS patients and obese women [9–11]. Furthermore, administration of kisspeptin in young healthy men caused increased GSIS assessed by intravenous glucose tolerance tests, suggesting a beneficial function of kisspeptin in enhancing insulin secretion in humans [15]. However, a study in nondiabetic obese adults reported that serum kisspeptin was inversely associated with an oral glucose tolerance test (OGTT)-derived indices of insulin secretion [13]. Moreover, the circulating kisspeptin level was greater in patients with type 2 diabetes (T2D) as compared with nondiabetic individuals [16].

In children, there is a period of transition through puberty during which insulin sensitivity is physiologically declined [17]. Kisspeptin, which plays a role in puberty initiation and energy balance and metabolism regulation, may be associated with glucose metabolism in obese children. In contrast to adult studies, the serum kisspeptin level was demonstrated to be positively correlated with BMI, waist circumference, and body weight in a group of obese and normal-weight Chinese children [18]. Nevertheless, data on serum kisspeptin levels in obese children and its associations with metabolic parameters and glucose metabolism are limited.

This study aimed to determine serum kisspeptin levels in obese children and study the associations between serum kisspeptin levels and glucose metabolism as well as metabolic parameters.

## 2. Materials and Methods

### 2.1. Subjects

There were 270 overweight and obese children enrolled. All of them were recruited from the outpatient clinic of the Department of Pediatrics, Faculty of Medicine Ramathibodi Hospital, Mahidol University, Bangkok, Thailand. Inclusion criteria were children with BMI standard deviation scores (SDSs) of greater than 1 according to the World Health Organization (WHO) BMI reference who were at least 10 years old or less than 10 years old but had entered puberty. Children who had syndromic obesity or chronic illness and received medications that interfered with glucose and lipid metabolism were excluded. The study was performed according to the Declaration of Helsinki and was approved by the Ethics Committee of the Faculty of Medicine Ramathibodi Hospital, Mahidol University (MURA2018/977, dated 9 January 2019). Written informed consent was obtained from all participants and their legal guardians.

### 2.2. Methods

This cross-sectional study collected clinical data including age, sex, pubertal status, weight, height, and waist circumference. Weight and height SDSs were determined using the National Standard Growth Curve Reference of the Ministry of Public Health, Thailand [19]. BMI was calculated by dividing weight (kilograms) by height (meters squared). The BMI SDS was calculated based on the WHO BMI reference [20]. The BMI SDS of greater than 1 to 2 was defined as overweight, and the BMI SDS of greater than 2 was defined as obesity. Waist circumference was measured at midway between the lowest ribs and superior border of the iliac crest with a nonelastic flexible tape. Owing to a lack of reference of waist circumference for Thai children, waist circumference percentile was assessed using the reference of Chinese children [21]. Pubertal status was determined using Marshall and Tanner's method [22, 23].

All patients underwent blood tests that included an OGTT and fasting serum kisspeptin, glycated hemoglobin (HbA1c), C-peptide, and lipid profile. Serum samples for kisspeptin measurement were kept at −80°C until analysis. Body composition analysis was performed using bioelectrical impedance, InBody 720® (BioSpace, Seoul, Korea). The SDS of body fat percentage was calculated using the previously published reference [24]. The serum kisspeptin level was measured using enzyme-linked immunosorbent assay (Cloud-Clone Corp., China) with the lower limit of detection of 13 pg/mL. The amount of analyzed plasma and replicates was 100 *μ*L. The intraassay and interassay coefficients of variation were <10% and <12%, respectively. Plasma glucose and serum insulin levels were measured using hexokinase/glucose-6-phosphate dehydrogenase method and chemiluminescent microparticle immunoassay, respectively. HbA1c measurement was performed using turbidimetric inhibition immunoassay, a certified and standardized method according to the American Diabetes Association guideline [25]. Other laboratory tests were analyzed using a routine automated analyzer.

In order to assess pubertal effects on serum kisspeptin levels, patients were divided into 2 groups according to their pubertal status (prepuberty and puberty). Each group was then divided into two subgroups according to their HbA1c levels (normal range, <5.7%, and prediabetes range, 5.7–6.4%). All data were analyzed and compared between the two subgroups of patients with the same pubertal status. Correlations between serum kisspeptin and other parameters were determined.

### 2.3. OGTT, Insulin Sensitivity, and *β*-Cell Function Assessment

An OGTT was performed at 08:00–09:00 after an 8- to 10-hour overnight fast. After collecting fasting blood samples for glucose and insulin level measurements, glucose solution at a dose of 1.75 grams per kilogram body weight (maximum 75 grams) was given orally within 5 minutes. Plasma glucose and serum insulin levels were then measured at 30, 60, 90, and 120 minutes after the ingestion of glucose solution.

Hyperinsulinemia (HI), which indicates insulin resistance, was characterized by having a fasting serum insulin level of ≥104 pmol/L, a 2-hour post-OGTT insulin level of ≥521 pmol/L, or a maximum insulin level during the OGTT of ≥1042 pmol/L [26]. Impaired fasting glycemia (IFG) was defined as a fasting plasma glucose (FPG) level of 5.6–6.9 mmol/L, and impaired glucose tolerance (IGT) was defined as a 2-hour plasma glucose level of 7.8–11.0 mmol/L. Diabetes was diagnosed if patients had either an FPG level of 7.0 mmol/L or greater or a 2-hour plasma glucose level of 11.1 mmol/L or greater [25].

To evaluate insulin sensitivity, whole-body insulin sensitivity index (WBISI) [27], homeostasis model assessment of insulin resistance (HOMA-IR) [28], and quantitative insulin sensitivity check index (QUICKI) [29] were determined. Insulinogenic index (IGI) and HOMA index (HOMA-*β*) [30] were calculated for assessment of *β*-cell function (insulin secretion). The following formulas were used with FI representing fasting serum insulin and *G* representing plasma glucose. Δ glucose 0–30 and Δ insulin 0–30 indicated the increments of glucose and insulin at 30 minutes of the OGTT, respectively:

- WBISI = 10,000/√FPG (mg/dL) × FI (*μ*IU/mL) × mean *G* (mg/dL) × mean insulin (*μ*IU/mL)
- HOMA-IR = (FPG (mmol/L) × FI (*μ*IU/mL))/22.5
- QUICKI = 1/(log FI (*μ*IU/mL) + log FPG (mg/dL))
- IGI = Δ insulin 0–30 (*μ*IU/mL)/Δ glucose 0–30 (mg/dL)
- HOMA-*β* = (20 × FI (*μ*IU/mL))/(FPG (mmol/L) − 3.5)

Additionally, the area under the curves (AUC) of plasma glucose and serum insulin was calculated using the trapezoidal method using the plasma glucose and serum insulin levels derived from the OGTT.

### 2.4. Statistical Analysis

The IBM SPSS Statistics for Windows, version 24.0 (IBM Corp., Armonk, NY, USA), and the STATA/SE for Windows, version 16.0 (StataCorp LLC, Texas, USA), were used for the analysis. Chi-squared tests were used to compare the categorical data. All continuous data were tested for the distribution using the Kolmogorov–Smirnov test. Non-normally distributed data were reported as median and interquartile range (IQR). The Mann–Whitney *U* test was applied for comparison of continuous data between two groups of patients. Differences among more than two groups were evaluated by the Kruskal–Wallis test. The median regression analysis was performed to determine associations between serum kisspeptin and other parameters. A *p* value of less than 0.05 was considered statistically significant.

## 3. Results

Median (IQR) age of 270 enrolled patients was 12.1 (10.9, 13.9) years. There were 127 males (47%) and 143 females (53%). Most children were pubertal with 106 (39%) and 138 (51%) of them having Tanner stages II-III and IV-V, respectively. The remaining 26 children (10%) were prepubertal. The median (IQR) BMI SDS of all children was 2.5 (2.1, 2.9). Forty-eight patients (18%) were overweight, and 222 patients (82%) were obese. The majority of children (*N* = 179, 66%) had a normal HbA1c level of <5.7%. Ninety-one patients (34%) had HbA1c levels between 5.7 and 6.4%, which were the prediabetes levels [25]. With regard to glucose metabolism status, 39 (14%), 164 (61%), and 67 (25%) patients had normal glucose tolerance (NGT), HI, and abnormal glucose tolerance (AGT), respectively. Among 67 patients with AGT, 4 patients had T2D, 3 had IFG, and the remaining 60 had IGT. Clinical characteristics and related parameters of all patients and each subgroup of pubertal status according to HbA1c ranges are shown in Table 1.

**Figure 1:**
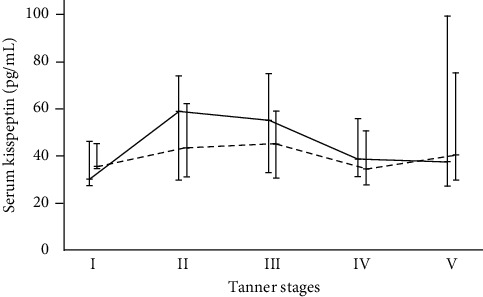
Serum kisspeptin levels across 5 genital Tanner stages in boys (*N*  = 127) and 5 breast Tanner stages in girls (*N* = 143) (*p* = 0.034 for boys and 0.730 for girls). Error bars represent median (horizontal line) and interquartile range (bar); the solid line represents boys; and the dashed line represents girls.

The median (IQR) serum kisspeptin level of all patients was 39.5 (30.1, 67.1) pg/mL. Serum kisspeptin levels were not different between boys and girls (boys: 41.7 (29.6, 69.7), girls: 39.2 (30.3, 64.0) pg/mL, *p* = 0.532). In addition, comparing between boys and girls of each Tanner stage, serum kisspeptin levels were not different. Serum kisspeptin levels were greatest in children with Tanner stages II-III (I: 31.4 (28.1, 48.6); II-III: 50.2 (30.7, 68.9); and IV-V: 38.5 (29.6, 68.5) pg/mL; *p* = 0.015). The significantly highest serum kisspeptin level was observed at Tanner stage II in boys (*p* = 0.034), but the finding was not reproduced in girls (Figure 1). No difference in serum kisspeptin levels was observed between overweight and obese groups (48.1 (31.8, 79.8) and 38.4 (29.6, 66.2) pg/mL, respectively; *p* = 0.203). Additionally, there was no significant difference in serum kisspeptin levels among three groups of glucose metabolism status (NGT: 41.5 (29.3, 69.7), HI: 38.8 (29.6, 66.0), and AGT: 43.5 (31.7, 72.4) pg/mL; *p* = 0.422). No significant interval changes of serum kisspeptin levels during the OGTT were observed in our pilot study (0 minute: 28.6 (27.2, 33.2), 30 minutes: 27.2 (27.0, 33.5), 60 minutes: 28.5 (26.9, 36.3), 90 minutes: 26.8 (26.8, 36.0), and 120 minutes: 26.8 (26.8, 33.5) pg/mL; *p* = 0.734).

Serum kisspeptin levels of children who had prediabetes HbA1c levels were significantly higher than those with normal HbA1c levels (51.9 (32.0, 68.4) and 36.7 (29.3, 64.0) pg/mL; *p* = 0.023). Although the BMI SDS, waist circumference percentile, and percentage of body fat SDS were comparable between the two HbA1c groups, patients with prediabetes HbA1c had the greater AUC of glucose, degree of insulin resistance, and proportion of children with AGT than those with normal HbA1c. Subgroup analysis according to pubertal status showed greater serum kisspeptin levels in children with prediabetes HbA1c levels only in the pubertal group (53.2 (33.9, 69.8) vs 37.8 (29.6, 67.5) pg/mL, *p* = 0.015) (Table 1). Notably, these pubertal children with two different HbA1c ranges had no differences in the BMI SDS, waist circumference percentile, and percentage of body fat SDS. Moreover, pubertal children with prediabetes HbA1c levels had the higher AUC of glucose and insulin, degree of insulin resistance, and proportion of patients with AGT than pubertal children with normal HbA1c. However, there was no significant difference in IGI, an index of first-phase insulin secretion [31]. In contrast with the findings in pubertal children, such findings were not demonstrated in the group of prepubertal children. Subgroup analysis of pubertal children according to gender revealed no statistically significant difference in serum kisspeptin levels between pubertal children with prediabetes and normal HbA1c ranges in both genders (boys: 58.7 (34.3, 88.0) vs 37.9 (29.7, 70.4) pg/mL, *p* = 0.055; girls: 50.7 (33.3, 66.9) vs 37.2 (28.7, 63.3) pg/mL, *p* = 0.146). However, there was a trend towards higher serum kisspeptin levels in both boys and girls with prediabetes HbA1c ranges. Small sample size of each subgroup could possibly explain the finding of having no statistically significant difference.

Associations between serum kisspeptin and clinical as well as metabolic parameters are shown in Table 2. Serum kisspeptin was positively associated with Tanner stages II-III (*β* = 18.37, *p* = 0.027) and HbA1c (*β* = 14.64, *p* < 0.001). After adjusting for puberty, HbA1c remained positively associated with serum kisspeptin (*β* = 12.87, *p* = 0.001). No correlations between serum kisspeptin and insulin secretion and insulin sensitivity indices were demonstrated.

## 4. Discussion

To the best of our knowledge, this is the first study that assessed serum kisspeptin levels in a relatively large number of obese children with different HbA1c ranges. By defining prediabetes state using the HbA1c level of 5.7–6.4%, approximately one-third of our obese children would be at high risk for developing diabetes. Our study demonstrated that obese children with prediabetes HbA1c had higher serum kisspeptin levels than those with normal HbA1c. Furthermore, serum kisspeptin was positively associated with HbA1c. In subgroup of pubertal children, higher serum kisspeptin levels were also demonstrated in children with prediabetes HbA1c levels, while there was no difference in prepubertal children. Pubertal effects on serum kisspeptin could potentially contribute to the difference. However, the limited number of prepubertal children in this study might prevent the identification of significance, if any. Theoretically, children with prediabetes HbA1c potentially have higher insulin secretion to compensate with insulin resistance. Among pubertal obese children in this study, comparable IGI and HOMA-*β* between those with normal and prediabetes HbA1c may reflect an impairment of first-phase insulin secretion in children with prediabetes HbA1c, which is similar to patients with IFG and IGT [31]. Pubertal obese children with prediabetes HbA1c also had lower insulin sensitivity (lower WBISI and QUICKI, but higher HOMA-IR), but higher total insulin secretion (higher AUC of insulin) as a compensatory mechanism [31], than those with normal HbA1c. Therefore, the finding of greater serum kisspeptin levels in pubertal patients with prediabetes HbA1c as compared with those with normal HbA1c might be related to the stimulatory effect of kisspeptin on insulin secretion to compensate for insulin resistance. Our findings might be partially supported by previous studies. Obese diabetic rats had a significant increase in hepatic kisspeptin levels as compared with rats without diabetes [8]. Additionally, a previous study in patients with morbid obesity and T2D had decreased serum kisspeptin levels and HbA1c together with decreased insulin secretion and increased insulin sensitivity following Roux-en-Y gastric bypass surgery [32]. Moreover, placental-derived kisspeptin was positively correlated with insulin secretion, suggesting a physiological adaptation to increased insulin resistance during pregnancy [33].

Despite the fact that individuals with AGT tend to have greater HbA1c as compared with those with NGT, this study only found higher serum kisspeptin levels in patients with prediabetes HbA1c levels as compared with patients with normal HbA1c, while there was no difference in serum kisspeptin levels between patients with AGT and NGT. In fact, HbA1c reflects average plasma glucose during the past few months, whereas glucose tolerance status reflects plasma glucose response to glucose loading at the time of OGTTs. Therefore, individuals who have AGT status are not identical to those having prediabetes HbA1c. This may explain higher serum kisspeptin levels in patients with prediabetes HbA1c, but not in patients with AGT.

This study did not demonstrate the associations between serum kisspeptin levels and indices of insulin sensitivity and insulin secretion, as well as glucose metabolism status in obese children. These findings were in contrast with those found in previous adult studies that showed association of kisspeptin and insulin sensitivity and GSIS [9–11, 13]. In PCOS women, serum kisspeptin was negatively correlated with HOMA-IR, an index of insulin resistance [9, 10]. In addition, a study in nonobese and obese women demonstrated a negative correlation between serum kisspeptin and HOMA-IR as well as fasting serum insulin levels [11]. Inverse correlation between serum kisspeptin and IGI was found in nondiabetic obese adults [13]. The difference between the findings of this study and the adult studies could be related to the study population. Abnormal glucose metabolism following the defective kisspeptin signalling was previously demonstrated as age-related emergence in mice studies. In young adult *Kiss1r* knock-out female mice, their glucose tolerance remained normal despite having increased adiposity [5, 6]. However, AGT was observed only later in adulthood [5, 6]. Owing to the fact that the majority of our studied patients were adolescents, associations between serum kisspeptin and indices of insulin sensitivity and insulin secretion as well as abnormal glucose metabolism may not yet be apparent. However, a significant relationship among these parameters might be observed subsequently in their adulthood.

The link between serum kisspeptin and obesity remains conflicting and its mechanism is yet to be elucidated. Previous adult studies demonstrated that serum kisspeptin was negatively associated with BMI in several population groups [9–14]. In contrast, some studies in both prepubertal and pubertal children showed a higher serum kisspeptin in obese girls as compared with those with normal weight [18, 34]. Neither significant association between serum kisspeptin and BMI nor significant difference in serum kisspeptin between overweight and obese children was found in this study.

In obese boys, difference in serum kisspeptin during pubertal progression was observed. Serum kisspeptin levels reached maximum at Tanner stage II and then gradually declined thereafter. Such pattern of serum kisspeptin levels during pubertal progression was different from that of Chinese boys whose serum kisspeptin levels continuously increased from Tanner stage I to stage V [18]. The Chinese study included both normal and obese children, whereas this study involved only overweight and obese children. Obesity might influence serum kisspeptin levels during the progression of puberty due to the disturbance of hypothalamic kisspeptin by inflammatory cytokines, leptin, insulin resistance, and elevated estrogen secondary to obesity [35].

We acknowledge some limitations of this study. First, healthy control children with normal BMI were not included for comparison. Second, the number of children with T2D was minimal, so the association, if any, could not be demonstrated. Third, the number of pubertal children in the subgroups according to gender was small and the number of prepubertal children was even smaller. Thus, statistical analysis and clinical application could be limited.

## 5. Conclusions

Serum kisspeptin levels in pubertal obese children with prediabetes HbA1c were greater than those with normal HbA1c. Serum kisspeptin was positively associated with HbA1c, but not with glucose metabolism status and indices of insulin sensitivity and insulin secretion.

## Figures and Tables

**Table 1:** Clinical characteristics and related parameters of all patients and each subgroup of pubertal status according to HbA1c.

Characteristics/parameters	All (*N* = 270)			Prepuberty (*N* = 26)			Puberty (*N* = 244)		
	Normal HbA1c (*N* = 179)	Prediabetes HbA1c (*N* = 91)	*p* value^*∗*^	Normal HbA1c (*N* = 17)	Prediabetes HbA1c (*N* = 9)	*p* value^†^	Normal HbA1c (*N* = 162)	Prediabetes HbA1c (*N* = 82)	*p* value^‡^
Age (years)	12.7 (11.1, 14.5)	11.8 (10.7, 12.9)	0.003	11.0 (10.4, 11.8)	10.7 (10.5, 11.7)	1.000	12.9 (11.3, 14.8)	11.9 (10.8, 13.1)	0.002
Male/female, *N* (%)	82/97 (46/54)	45/46 (49/51)	0.571	16/1 (94/6)	7/2 (78/22)	0.268	66/96 (41/59)	38/44 (46/54)	0.403
Weight SDS	4.9 (3.2, 6.0)	4.4 (3.8, 6.1)	0.533	2.9 (2.5, 3.9)	3.9 (3.2, 5.6)	0.029	5.0 (3.4, 6.1)	4.5 (3.8, 6.3)	0.876
Height SDS	0.9 (0.1, 1.8)	1.3 (0.4, 2.3)	0.039	0.4 (-0.3, 1.4)	1.1 (0.5, 1.5)	0.178	1.0 (0.1, 1.9)	1.3 (0.4, 2.3)	0.066
BMI SDS	2.6 (2.1, 2.9)	2.5 (2.2, 2.9)	0.834	2.6 (2.3, 2.9)	2.6 (2.3, 3.2)	0.609	2.6 (2.1, 2.9)	2.5 (2.1, 2.9)	0.917
Waist circumference percentile	128 (114, 148)	127 (116, 151)	0.473	115 (101, 120)	116 (102, 131)	0.293	130 (115, 150)	129 (118, 151)	0.658
Body fat percentage SDS	3.3 (2.7, 4.1)	3.4 (2.7, 4.0)	0.609	3.5 (3.3, 3.8)	3.6 (3.2, 4.0)	0.841	3.2 (2.6, 4.1)	3.4 (2.6, 4.0)	0.642
Kisspeptin (pg/mL)	36.7 (29.3, 64.0)	51.9 (32.0, 68.4)	0.023	34.3 (27.4, 50.9)	30.8 (28.7, 45.4)	0.893	37.8 (29.6, 67.5)	53.2 (33.9, 69.8)	0.015
C-peptide (nmol/L)	1.2 (0.9, 1.4)	1.3 (1.0, 1.6)	0.021	0.8 (0.7, 1.3)	1.1 (0.7, 1.2)	0.586	1.2 (0.9, 1.4)	1.3 (1.0, 1.7)	0.018
HbA1c (%)	5.4 (5.2, 5.5)	5.9 (5.8, 6.1)	<0.001	5.4 (5.2, 5.5)	5.8 (5.7, 6.0)	<0.001	5.4 (5.1, 5.5)	5.9 (5.8, 6.1)	<0.001
Fasting plasma glucose (mmol/L)	4.4 (4.3, 4.7)	4.6 (4.3, 4.9)	0.153	4.5 (4.4, 4.7)	4.8 (4.6, 5.1)	0.025	4.4 (4.3, 4.8)	4.5 (4.3, 4.8)	0.407
1-h plasma glucose (mmol/L)	7.4 (6.6, 8.6)	8.4 (7.2, 9.8)	<0.001	7.9 (6.7, 9.5)	9.4 (6.8, 10.2)	0.374	7.4 (6.5, 8.5)	8.3 (7.2, 9.7)	<0.001
2-h plasma glucose (mmol/L)	6.7 (5.7, 7.4)	7.3 (6.4, 8.0)	0.001	7.3 (6.3, 7.6)	6.8 (6.3, 7.8)	0.978	6.7 (5.7, 7.4)	7.3 (6.4, 8.0)	0.001
AUC glucose (h∙mmol/L)	13.9 (12.5, 15.3)	14.9 (13.4, 16.9)	0.001	15.1 (14.0, 17.0)	15.8 (13.6, 18.1)	0.535	13.7 (12.4, 15.2)	14.8 (13.2, 16.8)	0.001
Fasting plasma insulin (pmol/L)	108 (74, 152)	120 (89, 207)	0.015	81 (58, 152)	103 (55, 145)	0.609	110 (75, 152)	126 (90, 216)	0.015
1-h plasma insulin (pmol/L)	820 (566, 1340)	1070 (704, 1625)	0.019	763 (501, 1582)	1176 (425, 1715)	0.936	837 (570, 1311)	1063 (726, 1611)	0.015
2-h plasma insulin (pmol/L)	826 (478, 1361)	951 (617, 1688)	0.036	954 (640, 1340)	895 (494, 1320)	0.571	754 (452, 1374)	962 (634, 1717)	0.018
AUC insulin (h∙pmol/L)	1581 (1031, 2269)	1864 (1188, 2785)	0.067	1678 (1057, 2811)	1344 (831, 2778)	0.686	1571 (1028, 2242)	1867 (1220, 2907)	0.035
Glucose metabolism status, *N* (%)									
NGT	30 (17)	9 (10)		3 (18)	2 (22)		27 (17)	7 (8)	<0.001
HI	119 (66)	45 (49)		12 (70)	5 (56)	0.717	107 (66)	40 (49)	
AGT	30 (17)	37 (41)	<0.001	2 (12)	2 (22)		28 (17)	35 (43)	
WBISI	2.6 (1.7, 4.0)	2.0 (1.2, 3.0)	0.006	2.4 (1.6, 4.0)	2.7 (1.4, 5.5)	0.979	2.6 (1.7, 4.0)	2.0 (1.2, 2.9)	0.004
QUICKI	0.32 (0.31, 0.34)	0.32 (0.29, 0.33)	0.009	0.34 (0.31, 0.35)	0.32 (0.31, 0.37)	0.571	0.32 (0.31, 0.34)	0.31 (0.29, 0.33)	0.011
HOMA-IR	3.2 (2.0, 4.4)	3.7 (2.6, 6.5)	0.009	2.3 (1.8, 4.1)	3.3 (1.6, 4.5)	0.571	3.2 (2.0, 4.6)	3.9 (2.6, 6.8)	0.011
IGI	2.0 (1.3, 3.2)	1.8 (1.3, 3.0)	0.480	1.5 (1.0, 2.2)	1.5 (1.3, 1.8)	0.893	2.2 (1.3, 3.3)	1.8 (1.3, 3.4)	0.524
HOMA-*β*	318 (201, 447)	316 (222, 527)	0.371	282 (123, 449)	245 (108, 298)	0.269	318 (209, 450)	320 (229, 611)	0.172
Total cholesterol (mmol/L)	4.5 (4.0, 5.0)	4.5 (4.0, 5.0)	0.911	4.9 (4.3, 5.8)	4.8 (4.4, 5.5)	0.914	4.5 (3.9, 5.0)	4.4 (4.0, 4.9)	0.997
HDL-C (mmol/L)	1.2 (1.0, 1.3)	1.1 (1.0, 1.2)	0.042	1.3 (1.2, 1.4)	1.3 (1.1, 1.5)	0.686	1.1 (1.0, 1.3)	1.1 (1.0, 1.2)	0.031
LDL-C (mmol/L)	2.9 (2.4, 3.4)	2.9 (2.5, 3.5)	0.678	3.1 (2.7, 4.0)	3.1 (2.7, 3.5)	0.850	2.9 (2.3, 3.4)	2.9 (2.5, 3.5)	0.593
Triglyceride (mmol/L)	1.0 (0.7, 1.5)	1.1 (0.9, 1.5)	0.093	0.8 (0.6, 1.4)	1.1 (0.8, 1.6)	0.345	1.0 (0.7, 1.5)	1.1 (0.9, 1.5)	0.158

AGT, abnormal glucose tolerance; AUC, area under the curve; BMI, body mass index; HbA1c, glycated hemoglobin; HDL-C, high-density lipoprotein cholesterol; HI, normal glucose tolerance with hyperinsulinemia; HOMA-IR, homeostasis model assessment of insulin resistance; HOMA-*β*, HOMA index; IGI, insulinogenic index; LDL-C, low-density lipoprotein cholesterol; NGT, normal glucose tolerance without hyperinsulinemia; QUICKI, quantitative insulin sensitivity check index; SDS, standard deviation score; WBISI, whole-body insulin sensitivity index. Data are presented as median (IQR); ^*∗*^comparing between all children with normal HbA1c and prediabetes HbA1c; ^†^comparing between prepubertal children with normal HbA1c and prediabetes HbA1c; and ^‡^comparing between pubertal children with normal HbA1c and prediabetes HbA1c (Mann–Whitney *U* test for continuous data and chi-square test for categorical data).

**Table 2:** Correlations between serum kisspeptin and other parameters.

Parameters	*β*-Coefficient	95% confidence interval	*p* value
Age	−1.058	−3.131 to 1.014	0.316
Female	−2.520	−11.656 to 6.616	0.588
Tanner stages			
II-III	18.370	2.127 to 34.613	0.027
IV-V	6.810	−9.057 to 22.678	0.399

BMI SDS	−2.584	−8.024 to 2.856	0.350
Waist circumference percentile	0.057	−0.094 to 0.208	0.457
Body fat percentage SDS	−0.248	−5.689 to 5.194	0.928
C-peptide	2.707	−3.415 to 8.830	0.384
HbA1c	14.640	6.853 to 22.427	<0.001
Fasting plasma glucose	4.459	−4.232 to 13.151	0.313
AUC glucose	0.551	−1.017 to 2.120	0.489
AUC insulin	−0.002	−0.005 to 0.002	0.366
WBISI	0.944	−1.175 to 3.062	0.381
QUICKI	57.837	−68.757 to 184.432	0.369
HOMA-IR	−0.139	−1.561 to 1.282	0.847
IGI	−1.662	−4.368 to 1.045	0.228
HOMA-*β*	0.001	−0.006 to 0.007	0.824
Total cholesterol	−0.310	−5.973 to 5.353	0.914
HDL-C	−17.925	−35.953 to 0.103	0.051
LDL-C	−1.834	−7.942 to 4.275	0.555
Triglyceride	6.505	−0.380 to 13.390	0.064

AUC, area under the curve; BMI, body mass index; HbA1c, glycated hemoglobin; HDL-C, high-density lipoprotein cholesterol; HOMA-IR, homeostasis model assessment of insulin resistance; HOMA-*β*, HOMA index; IGI, insulinogenic index; LDL-C, low-density lipoprotein cholesterol; QUICKI, quantitative insulin sensitivity check index; SDS, standard deviation score; WBISI, whole-body insulin sensitivity index. Correlations were evaluated using median regression analysis.

## Data Availability

The data that support the findings of this study are available from the corresponding author upon reasonable request.
